# mAb Das-1 recognizes 3’-Sulfated Lewis A/C, which is aberrantly expressed during metaplastic and oncogenic transformation of several gastrointestinal Epithelia

**DOI:** 10.1371/journal.pone.0261082

**Published:** 2021-12-15

**Authors:** Jeffrey W. Brown, Koushik K. Das, Vasilios Kalas, Kiron M. Das, Jason C. Mills

**Affiliations:** 1 Division of Gastroenterology, Department of Medicine, Washington University in St. Louis, School of Medicine, St. Louis, Missouri, United States of America; 2 Washington University in St. Louis, School of Medicine, St. Louis, Missouri, United States of America; 3 Physician Scientist Training Program, Department of Medicine, McGaw Medical Center of Northwestern University, Chicago, Illinois, United States of America; 4 Division of Gastroenterology, Rutgers Robert Wood Johnson Medical School, New Brunswick, New Jersey, United States of America; 5 Department of Pathology and Immunology, Washington University in St. Louis, School of Medicine, St. Louis, Missouri, United States of America; 6 Department of Developmental Biology, Washington University in St. Louis, School of Medicine, St. Louis, Missouri, United States of America; Peter MacCallum Cancer Centre, AUSTRALIA

## Abstract

**Introduction:**

Multiple previous studies have shown the monoclonal antibody Das-1 (formerly called 7E_12_H_12_) is specifically reactive towards metaplastic and carcinomatous lesions in multiple organs of the gastrointestinal system (e.g. Barrett’s esophagus, intestinal-type metaplasia of the stomach, gastric adenocarcinoma, high-grade pancreatic intraepithelial neoplasm, and pancreatic ductal adenocarcinoma) as well as in other organs (bladder and lung carcinomas). Beyond being a useful biomarker in tissue, mAb Das-1 has recently proven to be more accurate than current paradigms for identifying cysts harboring advanced neoplasia. Though this antibody has been used extensively for clinical, basic science, and translational applications for decades, its epitope has remained elusive.

**Methods:**

In this study, we chemically deglycosylated a standard source of antigen, which resulted in near complete loss of the signal as measured by western blot analysis. The epitope recognized by mAb Das-1 was determined by affinity to a comprehensive glycan array and validated by inhibition of a direct ELISA.

**Results:**

The epitope recognized by mAb Das-1 is 3’-Sulfo-Lewis A/C (3’-Sulfo-Le^A/C^). 3’-Sulfo-Le^A/C^ is broadly reexpressed across numerous GI epithelia and elsewhere during metaplastic and carcinomatous transformation.

**Discussion:**

3’-Sulfo-Le^A/C^ is a clinically important antigen that can be detected both intracellularly in tissue using immunohistochemistry and extracellularly in cyst fluid and serum by ELISA. The results open new avenues for tumorigenic risk stratification of various gastrointestinal lesions.

## Introduction

The monoclonal antibody Das-1 has been used extensively to study metaplasia and cancer in numerous tissues over the last 30 years (Table 1). Das-1 shows broad reactivity in human fetal tissue; [1] however, in adults at homeostasis, expression is primarily restricted to biliary and colonic epithelium as well as skin [2]. Despite, the absence of reactivity in normal healthy tissues of the GI foregut, the epitope is reexpressed when these tissues undergo metaplasia that increases risk for cancer and when carcinomatous transformation occurs [3–19]. Thus, the epitope recognized by Das-1 fulfills the criteria for being a true oncofetal antigen. In addition to expression in human tissues, we have recently validated the utility of Das-1 in identifying high risk pancreatic cystic lesions in a large multicenter trial, where we demonstrated that a simple ELISA for Das-1 in cyst fluid outperforms all clinical guidelines in identifying pancreatic cysts harboring malignancy [7,8].

**Table 1:** Summary of the prior literature describing Das-1 reactivity after metaplasic and/or oncogenic transformation of adult tissues that do not natively express this antigen at homeostasis.

Tissue	Transformation	Reference
Bladder	Cancer	Pantuck *et al*., *J Urol* 1997;158:1722–7. PMID: 9334587
		Pantuck *et al*., *Br J Urol* 1998;82:426–30. PMID: 9772883
Esophagus	Barrett’s	Das *et al*., *Ann Intern Med* 1994;120(9):753–6. PMID: 7511878
		DeMeester *et al*., *Am J Gastroenterol* 2002;97(10):2514–23. PMID: 12385432
		Hahn *et al*., *Am J Surg Pathol* 2009;33(7):1006–15. PMID: 19363439
Lung	Cancer	Deshpande *et al*., *Pathobiology* 2002;70(6):343–7. PMID: 12865630
Pancreas	Cancer	Das *et al*., *Gut* 2014;63(10):1626–34. PMID: 24277729
		Das *et al*., *Gastroenterology* 2019;157(3):720–30. PMID: 31175863
		Das *et al*., *Hum Pathol*. 2021;111:36–44. PMID: 33524436
		Heidarian et al., J Am Soc Cytopathol. 2021;10:249–54. PMID: 33541830
Small Bowel	Adenoma	Onuma *et al*., *Am J Gastroenterol* 2001;96(8):2480–5. PMID: 11513194
Stomach	Intestinal-Type Metaplasia	Glickman *et al*., *Am J Surg Pathol* 2001;25(1):87–94. PMID: 11145256
		DeMeester et al., Am J Gastroenterol 2002;97(10):2514–23. PMID: 12385432
		Mirza *et al*., *Gut* 2003;52(6):807–12. PMID: 12740335
		Piazuelo *et al*., *Mod Pathol* 2004;17(1):62–74. PMID: 14631367
		Watari *et al*., *Int J Cancer* 2012;130(10):2349–58. PMID: 21732341
Stomach	Cancer	Mirza *et al*., *Gut* 2003;52(6):807–12. PMID: 12740335
		O’Connell *et al*., *Arch Pathol Lab Med* 2005;129(3):338–47. PMID: 15737028
		Feng *et al*., *Exp Ther Med* 2013;5(6):1555–8. PMID: 23837030
		Kawanaka *et al*., *Br J Cancer* 2016;114(1):21–9. PMID: 26671747
		Watari *et al*., *Int J Cancer* 2012;130(10):2349–58. PMID: 21732341

In this study, we aim to identify the oncofetal antigen recognized by mAb Das-1 that has been used as a biomarker for high-risk metaplasia and cancer across numerous tissues in both histology as well as body fluids (serum and pancreatic cyst fluid). Here, using chemical deglycosylation, a comprehensive glycan array, and validation by inhibition of a direct ELISA, we demonstrate that the clinically important epitope of Das-1 is 3’-Sulfo-Le^A/C^.

## Results

**Fig 1:**
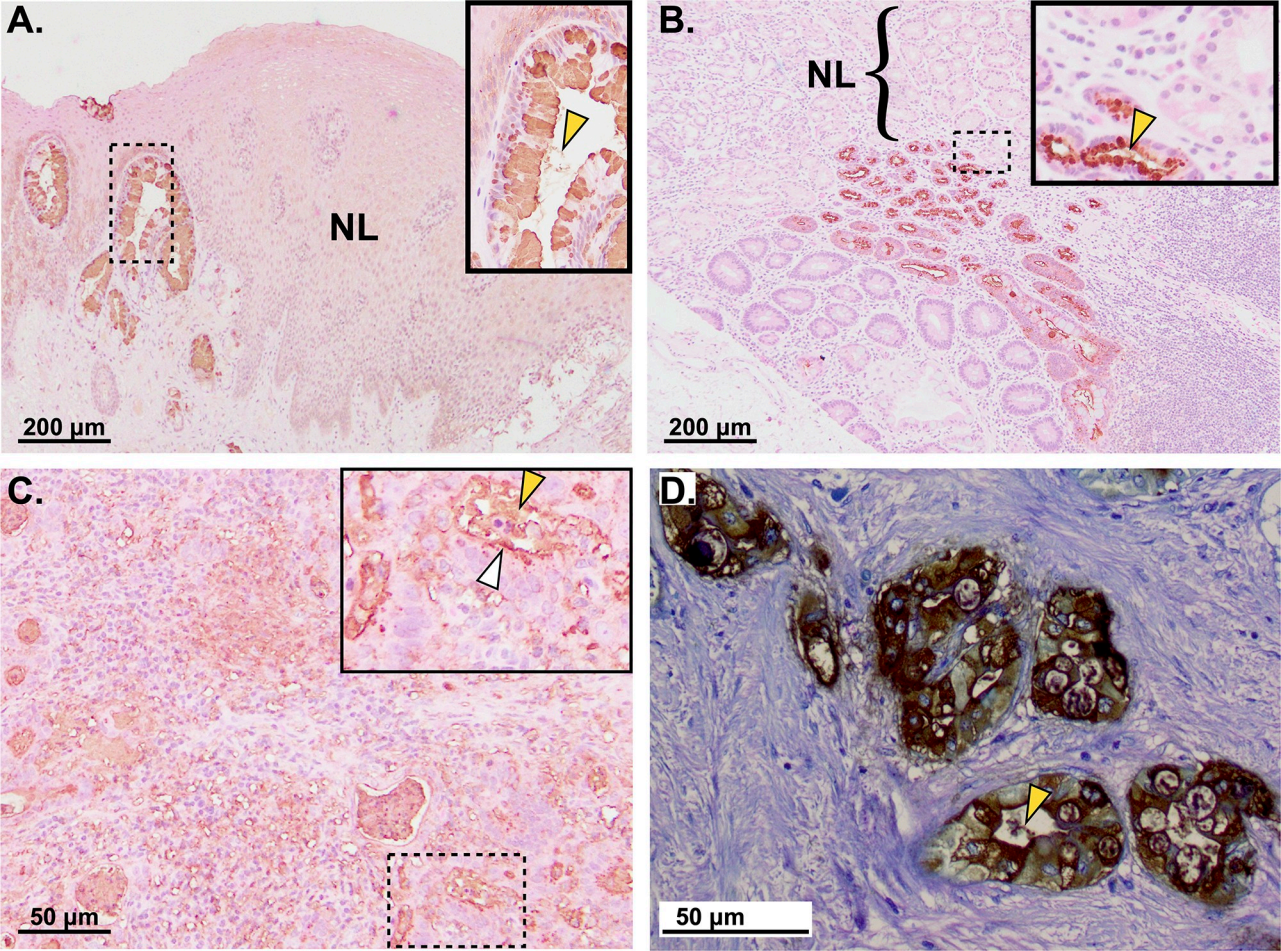
Unlike normal tissue, metaplastically and oncogenically transformed foregut tissues become reactive to mAb Das-1. Immunohistochemistry of **A**. Barrett’s Esophagus, **B**. Intestinal Metaplasia of the Stomach (from tissue adjacent to gastric cancer), **C**. Gastric Cancer, **D**. Pancreatic Ductal Adenocarcinoma. Scale bars presented in bottom left corner of each panel. Bracket labeled “NL” to highlight the absence of staining in either the esophageal squamous tissue or normal stomach; contrast with incomplete intestinal-type metaplasia, which expresses 3’-Sulfo-Le^A^. Insets show higher-magnification of boxed areas. White arrowhead: Das-1 staining at a cell apex; yellow arrowhead: 3’-Sulfo-Le^A^ that has been secreted into the extracellular space.

Immunohistochemistry of foregut metaplasias and cancers demonstrates that mAb Das-1 reactive material is expressed both intracellularly and is secreted (Fig 1). The latter phenomenon explains why it is detectable in extracellular fluid adjacent to high-grade dysplasia and cancer [7,8].

**Fig 2:**
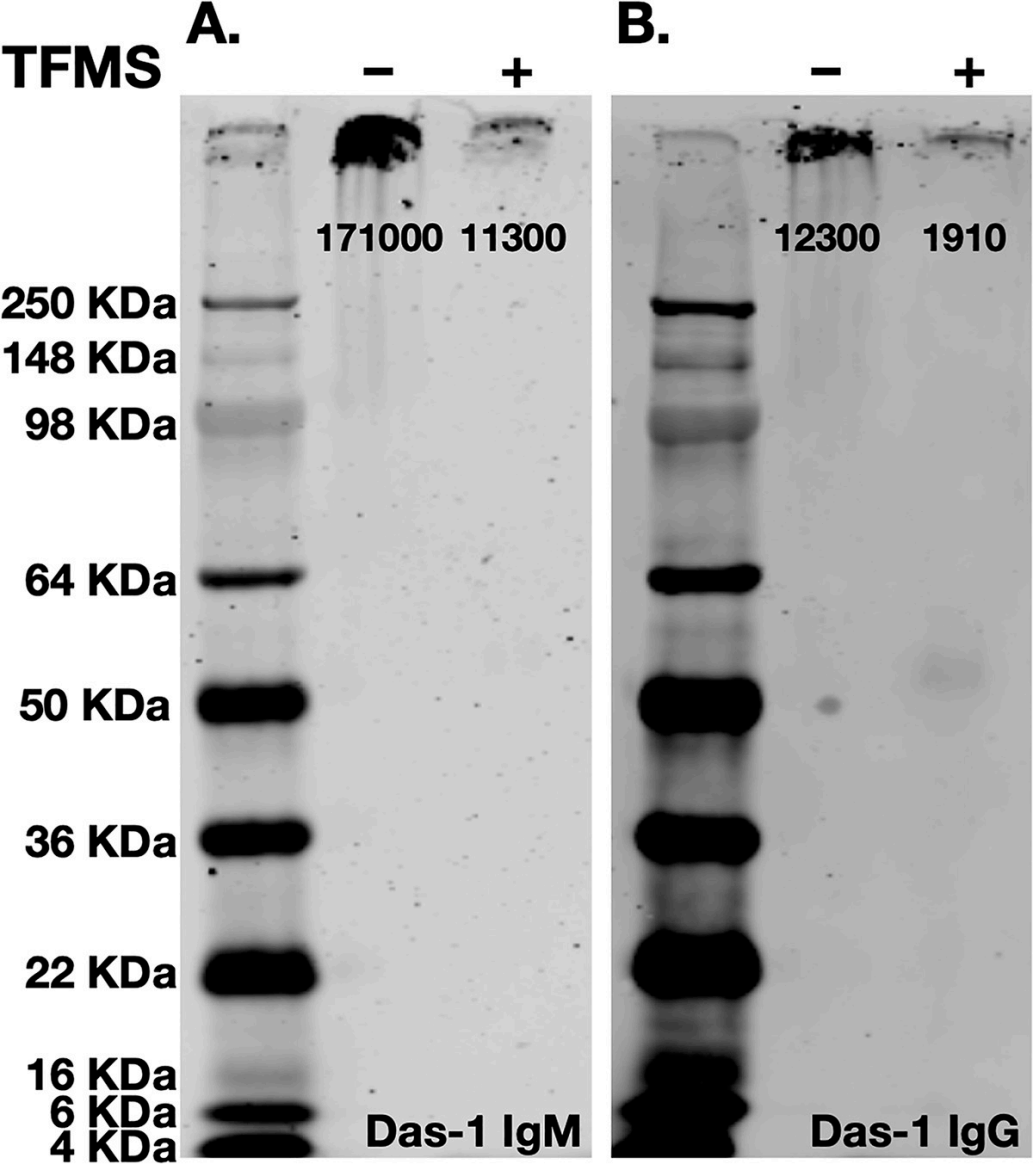
Das-1 IgG and IgM recognize a glycosylation epitope. Chemical deglycosylation of the antigen results in near complete loss of signal as measured by western blot analysis using (**A**) Das-1 IgM and (**B**) Das-1 IgG. Quantification of band intensity is presented below each band.

**Fig 3:**
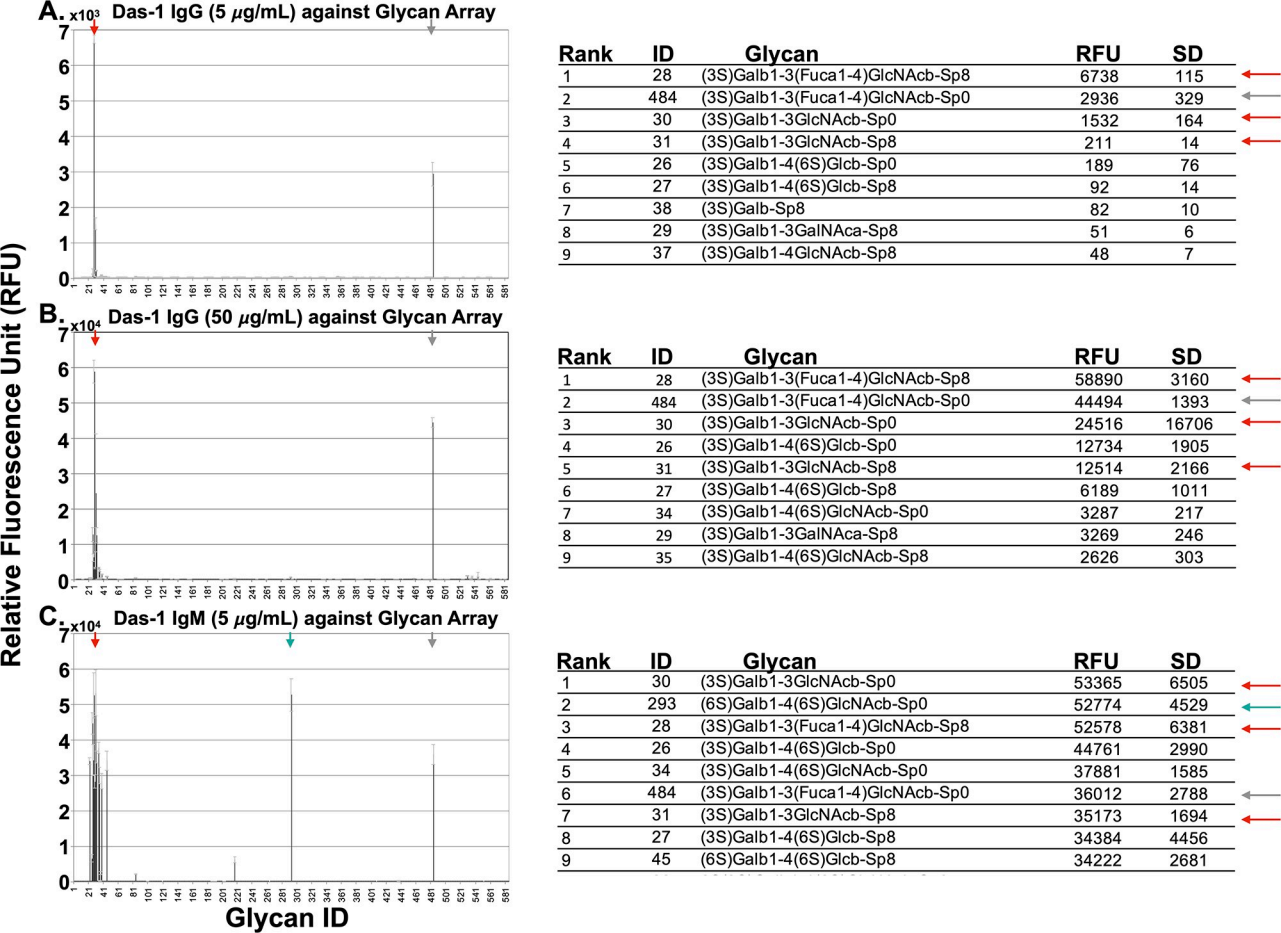
The results of the glycan arrays. Das-1 IgG (at 5 & 50 μg/mL) and Das-1 IgM (at 5 μg/mL) are plotted in A, B, and C respectively as the average relative fluorescence units (of 6 technical replicates) plus/minus standard deviation. The top 9 glycans for each arrays are listed to the right of each figure. Colored arrows emphasize that Das-1 IgG and Das-1 IgM recognize the same set of glycans. The complete data sets are provided in S1 Dataset (5 μg/mL IgG), S2 Dataset (50 μg/mL IgG), and S3 Dataset (5 μg/mL IgM) and are available for download on the Consortium for Functional Glycomics website (www.functionalglycomics.org).

Chemical deglycosylation with trifluoromethanesulfonic acid (TFMS) of a source of concentrated antigen recognized by Das-1 (media conditioned by the LS180 cell line, see Method section) resulted in near complete loss of Das-1 binding in western blot analysis (93% and 85% as measured by IgM and IgG, respectively; Fig 2) indicating the Das-1 epitope depended on glycans. Like most glycoproteins, the mucin used here contains a heterogeneous population of glycans [20]. Thus, we determined glycan specificity of the Das-1 IgM and Das-1 IgG antibodies against a comprehensive array of 584 glycans. Both Das-1 IgM and Das-1 IgG preferentially recognized Le^A/C^ that had been sulfated at the 3’ site of galactose (Fig 3). Thus, 3’-Sulfo-Galβ(1–3)GlcNAc (3’-Sulfo-Le^C^) appears to be the fundamental epitope recognized by the Das-1 antibody and that the α(1–4) linked fucose in 3’-Sulfo-Le^A^ likely modestly increases affinity. The antibody also recognizes some disulfated glycans, albeit with lower apparent affinity. The antibodies display little-to-no affinity for the non-sulfated, sialylated, or 6’-mono-sulfated counterparts, which are listed as pertinent negatives below the highest ranked hits (S1 Fig). Recognition of the epitope was also independent of net charge, as Mannose-6-Phosphate, another negatively charged sugar, was not recognized by either antibody (S1 Fig). Relative to the IgG, the IgM isotype had similar epitope specificity but had detectable affinities against broader range of glycans (Fig 3), as might be expected due to the greater avidity of its pentameric quaternary structure.

**Fig 4:**
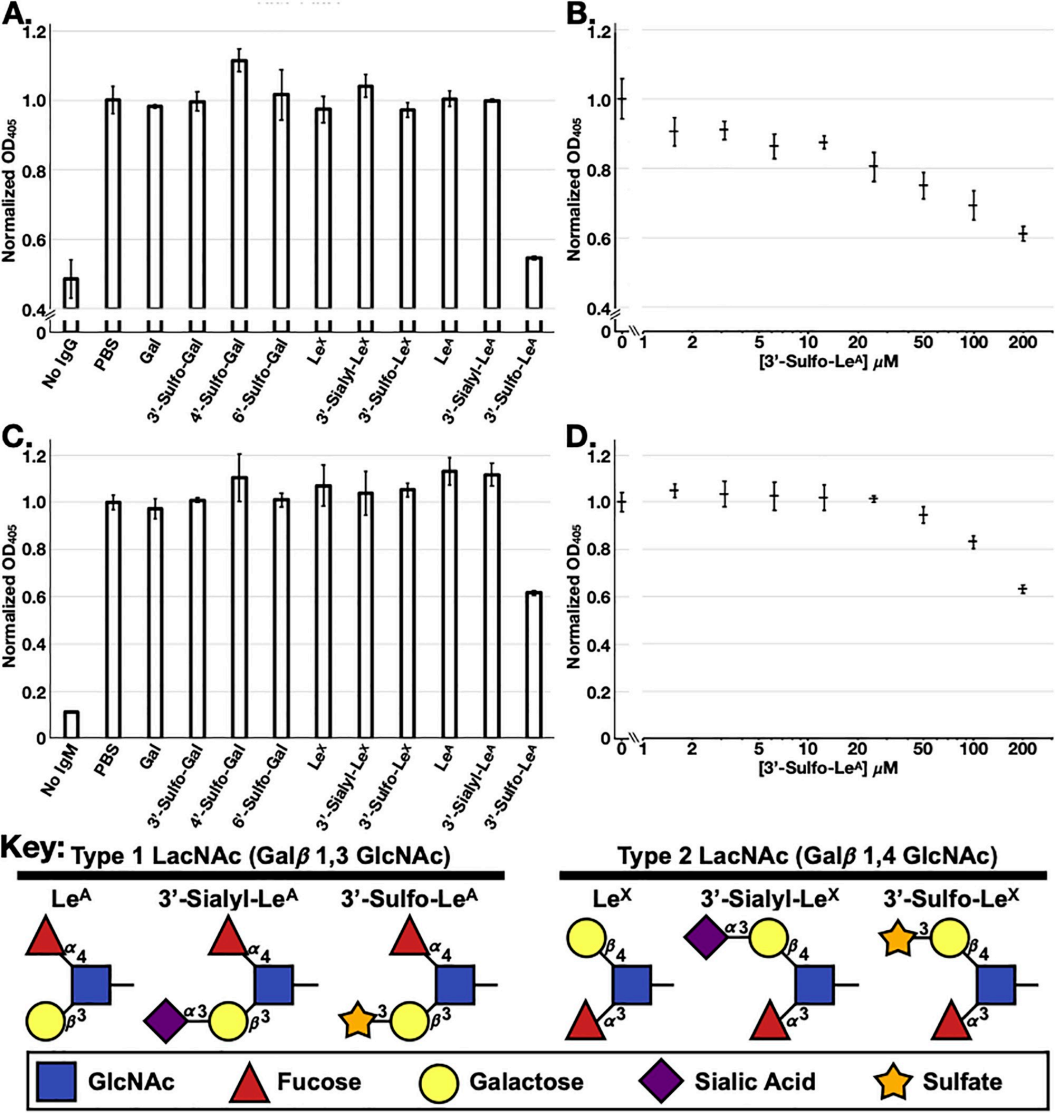
Das-1 IgG and IgM specifically recognize 3’-Sulfo-Le^A^. Direct ELISA using Das-1 IgG (**A**) or Das-1 IgM (**C**) in the absence or presence of several free glycans in solution at 200 μM. Direct ELISA using Das-1 IgG (**B**) or Das-1 IgM (**D**) against a titration series of 3’-Sulfo-Le^A^. Data reported as average ± standard deviation of three technical replicates normalized to the reaction without glycans (PBS). **Key**: Schematic diagram of the relevant Lewis antigens is provided for reference.

To confirm the epitope specificity, we performed a direct ELISA using Das-1 against the heterogeneously glycosylated high molecular weight mucin carrying the antigen and found that both Das-1 IgG and IgM were inhibited by 3’-Sulfo-Le^A^, in a dose-dependent manner (Fig 4), and neither the sialylated (3’-Sialyl-Le^A^; i.e. Ca19-9) nor unsulfated adducts were able to inhibit the reaction. Due to the high avidity of pentameric IgM (10 antigen binding sites) against mucins containing numerous glycosylation epitopes, we were only able to achieve 46% inhibition at 200 μM of the freely diffusing glycans compared to the 88% inhibition we achieved with the Das-1 IgG at the same concentration. The IC_50_ for IgG in this experiment was 48.9 μM. Despite only differing by the Galactose-GlcNAc-fucose arrangement (Fig 4 Key), Le^X^ (type II) adducts were not able to competitively inhibit Das-1 binding in the ELISA. Further, the assay was not inhibited by sulfated galactose in the absence of the adjacent GlcNAc in Le^A/C^ (Fig 4). Thus, both the affinity and inhibitory studies presented here are consistent with 3’-Sulfo-Le^A/C^ being the epitope recognized by both Das-1 IgG and IgM.

## Discussion

Aberrant glycosylation patterns (especially acidic modification including sialylation and sulfation) have been identified in numerous types of cancer, and probing for neo-glycosylation epitopes is a common clinical practice used to (1) detect cancer, (2) monitor therapeutic response, and/or (3) evaluate for recurrence. However, typically the utility of these glycosylation epitopes is restricted to a small set of cancers (e.g. CA19-9 for pancreatic cancer). In contrast, 3’-Sulfo-Le^A/C^ appears to be aberrantly expressed among numerous pre-neoplastic lesions and cancers (Fig 1, Table 1).

Sulfation, much like phosphorylation, is a posttranslational modification that adds a negatively charged moiety and can be used to regulate cellular processes. Sulfate groups can be detected using high-iron diamine staining; however, this technique has been removed from most commercial laboratories due to toxicity concerns [21]. Furthermore, this technique is specific only to the reactive sulfate group and not the glycan carrying this moiety. Since there are no commercially available lectins or antibodies currently available that are specifically reactive to glycans carrying a terminal sulfate, this posttranslational modification is poorly understood [22,23]. In an attempt to study sulfation, Rick Cummings’ group recently developed a novel sea lamprey variable lymphocyte receptor reactive to 3’-Sulfo-Le^X^ and characterized expression in a survey of normal adult tissue [23]. Other groups have used recombinantly expressed proteins like selectins; however, these lectins recognize terminally sialylated glycans in addition to those with terminal sulfates [22]. Thus, in addition to the diagnostic utility of this antibody, the specific reactivity towards 3’-Sulfo-Le^A/C^ that we reported here for mAb Das-1 provides a unique opportunity to study the cellular consequences of expressing this poorly understood post-translational modification.

This study is not without limitations. First, the high molecular weight mucins carrying 3’-Sulfo-Le^A/C^ are extremely difficult to electrophoretically resolve due to their extremely large size and heterogeneity in glycosylation as well as potentially protein carriers (e.g. compare Fig 1 to Issa *et al*., 2011 [24] whom used a different, historic antibody against 3’-Sulfo-Le^A^). Second, it is possible that despite being very comprehensive that the glycan array was lacking an epitope for which the Das-1 antibody has even greater affinity than 3’-Sulfo-Le^A/C^. Third, because 3’-Sulfo-Le^C^ is not commercially available, we were not able to directly test whether the Das-1 antibodies could be inhibited by this sugar in solution. We have provided indirect evidence for interaction for interaction with 3’-Sulfo-Le^C^ with murine models of oncologic progression. In mice, *Fut3* (the only enzyme that can add an α(1–4) linked fucose) is a pseudogene [25] and, as a consequence, mice are only able to synthesize 3’-Sulfo-Le^C^ and not 3’-Sulfo-Le^A^. Das-1 reactivity towards murine models of gastric intestinal metaplasia (data reviewed, but not shown) and pancreatic cancer (S2 Fig) demonstrate that reactivity phenocopies human disease [15,26,27].

3’-Sulfo-Le^A^ has been implicated in diverse cellular processes. For example, swallowed salivary 3’-Sulfo-Le^A^ on Muc5B has been shown to be a potent ligand for the gastric pathogen *H*. *pylori* [28]. The bacterial receptor for 3’-Sulfo-Le^A^ is believed to be neutrophil-activating protein (NapA) [29,30], which is invariably expressed across human strains of *H*. *pylori* [31]. The importance of NapA as a major virulence factor has been demonstrated in murine models: vaccination of mice with recombinantly expressed NapA provides protection against *H*. *pylori* challenge, which is consistent with anti-NapA antibodies being present in the majority of people infected with *H*. *pylori* [32]. Thus, swallowed salivary 3’-Sulfo-Le^A^ may serve as a decoy to saturate this virulence factor and limit *H*. *pylori* entry into the gastric glands. 3’-Sulfo-Le^A^ has been demonstrated to be a potent ligand for selectins (*e*.*g*. E-Selectin [33,34], L-Selectin [35–37], and P-Selectin [36]). Further, it has also been shown to bind proteins on macrophages (cysteine-rich domain of the macrophage mannose receptor [38]) as well as dendritic cells (dendritic cell immunoreceptor [39]). It remains to be determined why tumors of the foregut invariably express and secrete this 3’-Sulfo-Le^A/C^: whether it serves an intracellular function, is to avoid immune surveillance, or to modulate the microbiome.

The progression from normal tissue to metaplasia to cancer has arguably been best described in the stomach by seminal work of Pelayo Correa and others [40–43]. The pre-cancerous state of chronic atrophic gastritis is characterized by appearance of metaplastic cells deep in the gastric glands [44]. Such Spasmolytic Polypeptide Expressing Metaplasia (SPEM) or pseudopyloric metaplasia cells express Sialyl-Le^X^ antigens that promote binding of the pro-inflammatory, carcinogenic bacteria *H*. *pylori* [45,46]. Expression of these sialomucins within the columnar epithelial cells is also a defining feature of Type II, incomplete, intestinal-type metaplasia in the stomach [21]. Transition from sialylation to sulfation is the sole feature distinguishing Type II from Type III gastric intestinal metaplasia with the latter being associated with increased risk for progression to cancer [21,47–49]. Consistent with our data, another group has historically generated an antibody (91.9H) that recognizes 3’-Sulfo-Le^A^ and is reactive with Barrett’s esophagus [50] and GIM [51]; and phenocopies high iron diamine staining for sulfation in Barrett’s and GIM [51]. This antibody recognizes this antigen only in the context of a tetra- or penta-saccharide [52], while here we exclusively used trisaccharides and thus demonstrate that Das-1 recognizes the terminal 3’-Sulfo-Le^A^ trisaccharide and does not require other adjacent sugars.

Uncovering the diagnostically important 3’-Sulfo-Le^A^ modification has important implications. For one, new technologies for specifically detecting this glycan (*e*.*g*. mass spectroscopy [53]) may lead to even greater sensitivity in diagnosis of metaplasia and cancer at earlier stages and in a wider variety of fluids and tissues. Moreover, reproducibility of the Das-1 sandwich ELISA [7,8] for clinical laboratory applications may be improved by using pure 3’-Sulfo-Le^A^ as a standard as opposed to the current practice for Das-1: using antigen concentrated from a colon cancer cell line [7,8].

It remains to be determined (1) *why* metaplastic and cancerous tissue of the GI foregut ubiquitously express this antigen, (2) the *necessity* of this epitope for metaplastic and oncogenic transformation, (3) what proteins or lipids carry this epitope, and (4) the molecular mechanism by which this epitope is released in pancreatic cyst fluid [7,8] as well as in the serum of individuals with cancer [53,54]. If the cellular processes annotated by these 3’-Sulfo-Le^A^ antigens confer a proliferative or survival advantage to cancer then specifically inhibiting the sulfation reaction may provide a novel therapeutic strategy for these lethal cellular transformations.

## Methods

For western blots, lyophilized antigen derived from media conditioned by the LS180 colon cancer cell line (ATCC CL-187) [7] was deglycosylated using anhydrous trifluoromethanesulfonic acid (TFMS) per manufacturer (Sigma-Aldrich, USA) protocol. Briefly, 140 μL of TFMS and 15μL of anisole were added to lyophilized mucin for 3 hours at 2-8C. 4 μL of Bromophenol Blue was added to follow the neutralization reaction, which was accomplished by adding 60% pyridine solution in a dropwise fashion in a methanol-dry ice bath. Following the deglycosylation reaction, both control and TFMS treated antigen were diluted to the same final volume with Laemmli buffer with 50 mM DTT prior to being applied to the gel. The control reaction was treated in an identical fashion (lyophilization, anisole, and pyrimidine) to the experimental condition; the only difference was that TFMS was omitted. Western blot was performed using nitrocellulose blocked with 5% BSA in PBS. Das-1 IgM or IgG were used at 1 μg/mL and Goat anti-Mouse IgM (LiCOR 926–32280) or Donkey Anti-Mouse IgG (LiCOR 926–32212) were used at 1:5000 and imaged on a Odyssey CLX.

Both the original Das-1 IgM as well as Das-1 IgG (developed from a hybridoma that had undergone spontaneous isotype switch and thus with identical *in vivo* reactivity) were assayed against a comprehensive array of 584 glycans provided by the National Center for Functional Glycomics. Briefly, the array was generated from a library of natural and synthetic mammalian glycans with amino linkers printed onto N-hydroxysuccinimide (NHS)-activated glass microscope slides forming covalent amide linkages [55]. The glycan spotting concentration was 100 μM. 6 technical replicates were performed for each antibody. Detailed methods section is available at https://ncfg.hms.harvard.edu/protocols/glycan-binding-assay-unlabeled-monoclonal-antibody. Here, Das-1 IgM was tested at 5 μg/ml and Das-1 IgG at 5 μg/ml and 50 μg/ml with 1% BSA (Boval LY-0081) in 20 mM Tris pH 7.4, 150 mM NaCl, 2 mM CaCl_2_, 2 mM MgCl_2_, 0.05% Tween-20 against the array for 1 hour at room temperature. Secondary anti-mouse IgM (488) or anti-Mouse IgG (488) were used at 5 μg/ml in the same buffer and conditions. The Das-1 IgM and IgG antibodies were provided to CFG and as a fee-for-service and the analysis against the glycan array was completed blinded.

Epitope specificity was confirmed by ELISA of wells coated with antigen (0.5 μg/well), incubated in PBS overnight at 4ºC, blocked with 1% BSA (Sigma A7906) in PBS, and then incubated with 2.5 μg of either Das-1 IgG or Das-1 IgM ±200 μM of the respective carbohydrate in PBS. The reactions were developed after incubation with alkaline phosphate-conjugated anti-mouse IgG (Sigma A1418) or IgM (Sigma A9688) at 1:20000 in PBS with 1% BSA and absorption at 405 nm measured after adding phosphatase substrate (Sigma S0942) in 0.001M MgCl_2_, 0.05M Na_2_CO_3_ pH 9.6. Separate assays measured effects of competitive inhibition with 0 to 200 μM 3’-Sulfo-Le^A^. After coating the wells with antigen, all incubations were one hour in duration and performed at room temperature and the wells were washed three times with PBS containing 0.1% Tween-20 between each step.

The IC_50_ for IgG titration curve was calculated using Eq 1, where Abs is ELISA absorption at 405nm, [3’-SulfoLe^A^] is the concentration of small molecule inhibitor, Max was set to 1 and Min to 0.485 (no IgG control, Fig 4A). The IC_50_ and cooperativity were simultaneously refined via minimizing least squares using Excel’s Solver add-in. IC_50_ for IgG was 48.9 μM and cooperativity was 0.585. Identical analysis of the IgM inhibition study (with Min set to 0.112) suggests that the IC_50_ is ~239 μM and the transition more cooperative at ~1.78; however, these values should be viewed as estimates because the freely diffusible 3’-Sulfo-Le^A^ only partially inhibited the high-order avidity between pentameric IgM and multivalent glycosylated mucins (46% inhibition at 200 μM).


$$Abs=1-\frac{Max-Min}{1+{\left(\frac{{IC}_{50}}{\left[3^{\prime }Sulfo{Le}^{A}\right]}\right)}^{Cooperativity}}$$

Immunohistochemistry of paraffin embedded tissue was performed in a standard fashion. Briefly dewaxing was accomplished with Histoclear and the slides were hydrated using an ethanol series. Antigen retrieval was with a pressure cooker (5 minutes) in 10 mM citrate, pH 6.0. Blocking using 2% BSA (Sigma A7906). Tissue was probed with 1 μg/mL Das-1 IgM in PBS with 2% BSA and 0.2% Triton X-100 overnight at 4C. Biotinylated goat anti-Mouse IgM (Vector Lab BA-2020) was used as a secondary (1:200 dilution in PBS with 2% BSA and 0.2% Triton X-100 for 1 hour at room temperature). Vectastain ABC Elite kit (Peroxidase; Vector PK-6100) diluted in in PBS with 2% BSA and 0.2% Triton X-100 for 1 hour at room temperature followed by 3.3’-Diaminobenzidine (DAB) for 1 minute at room temperature.

JWB & JCM are the guarantor of this work and, as such, had full access to all of the data in the study and takes responsibility for the integrity of the data and the accuracy of the data analysis.

## Supporting information

S1 Fig In depth presentation of pertinent positive and negative glycan results. Das-1 IgG (at 5 & 50 μg/mL) and Das-1 IgM (at 5 μg/mL) are plotted logarithmically in A, B, and C respectively as the average relative fluorescence units (of 6 technical replicates) plus/minus standard deviation. The top 20 glycans for each arrays are listed below each array along with pertinent negative results. The complete data sets are provided in S1 Dataset (5 μg/mL IgG), S2 Dataset (50 μg/mL IgG), and S3 Dataset (5 μg/mL IgM) and are available for download on the Consortium for Functional Glycomics website (www.functionalglycomics.org).
(PDF)

S2 Fig Das-1 reactivity in a murine model of pancreatic cancer progression phenocopies human pathology. Das-1 is not reactive to (A) normal pancreata or (B) acinar-to-ductal metaplasia; however, demonstrates a (C) variegated reactivity towards high-grade Pan-IN, which becomes confluent in (D) pancreatic ductal carcinoma (PDAC) and (E) invasive PDAC. This pattern phenocopies what we have observed our survey of human pancreatic cancer progression (Das *et al*. (2021) Human Pathology 111: 36–44).
(TIFF)

S1 Dataset Complete glycan array dataset of Das-1 IgG (5 ug/uL) against the CFG glycan array. This dataset will also be released for public download at www.functionalglycomics.org.
(XLSX)

S2 Dataset Complete glycan array dataset of Das-1 IgG (50 ug/uL) against the CFG glycan array. This dataset will also be released for public download at www.functionalglycomics.org.
(XLSX)

S3 Dataset Complete glycan array dataset of Das-1 IgM (5 ug/uL) against the CFG glycan array. This dataset will also be released for public download at www.functionalglycomics.org.
(XLSX)

S1 Raw images (TIFF)

## Abbreviations

BSA: Bovine Serum Albumin
DAB: 3.3’-Diaminobenzidine
DTT: Dithiothreitol
ELISA: Enzyme Linked Immunosorbent Assay
Gal: Galactose
GlcNAc: N-Acetylglucosamine
Le^A^: Lewis A
Le^C^: Lewis C
Le^X^: Lewis X
PBS: Phosphate Buffered Saline
TFMS: Trifluoromethanesulfonic Acid
