# Supplementary material for: mAb Das-1 recognizes 3’-Sulfated Lewis A/C, which is aberrantly expressed during metaplastic and oncogenic transformation of several gastrointestinal Epithelia
Source: PLoS One. 2021 Dec 15;16(12):e0261082. doi: 10.1371/journal.pone.0261082 (PMC8673611; doi:10.1371/journal.pone.0261082)
Supplement: S1 Fig — Das-1 IgG (at 5 & 50 μg/mL) and Das-1 IgM (at 5 μg/mL) are plotted logarithmically in A, B, and C respectively as the average relative fluorescence units (of 6 technical replicates) plus/minus standard deviation. The top 20 glycans for each arrays are listed below each array along with pertinent negative results. The complete data sets are provided in S1 Dataset (5 μg/mL IgG), S2 Dataset (50 μg/mL IgG), and S3 Dataset (5 μg/mL IgM) and are available for download on the Consortium for Functional Glycomics website (www.functionalglycomics.org). (PDF) [file pone.0261082.s001.pdf]

A.

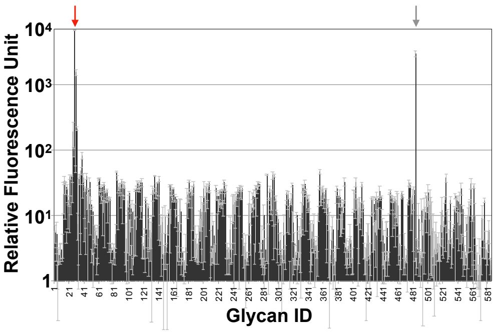

B.

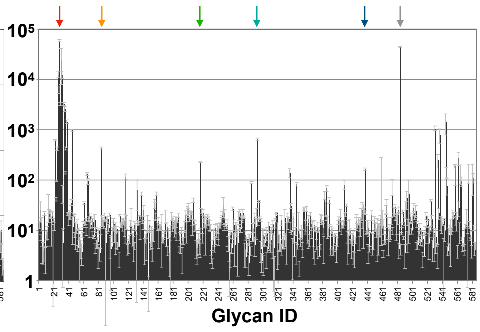

C.

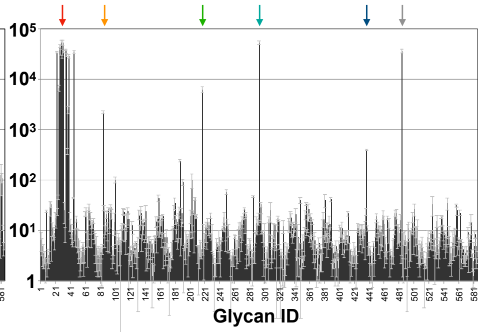

IgG Affinities (5 μg/mL)

Rank	ID	Glycan	RFA	SD
1	28	(3S)Galb1-3(Fuca1-4)GlcNAcb-Sp8	6738	115
2	484	(3S)Galb1-3(Fuca1-4)GlcNAcb-Sp0	2936	329
3	30	(3S)Galb1-3GlcNAcb-Sp0	1532	164
4	31	(3S)Galb1-3GlcNAcb-Sp8	211	14
5	26	(3S)Galb1-4(6S)Glcb-Sp0	189	76
6	27	(3S)Galb1-4(6S)Glcb-Sp8	92	14
7	38	(3S)Galb-Sp8	82	10
8	29	(3S)Galb1-3GalNAca-Sp8	51	6
9	37	(3S)Galb1-4GlcNAcb-Sp8	48	7
10	356	Fuca1-2Galb1-4(Fuca1-3)GlcNAcb1-2Mana1-6(Fuca1-2Galb1-4(Fuca1-3)GlcNAcb1-2Mana1-3)Manb1-4GlcNAcb1-4GlcNAb-Sp20	47	3
11	84	(3S)Galb1-4(Fuca1-3)Glcb-Sp0	47	1
12	285	Galb1-3GlcNAcb1-3Galb1-3GlcNAcb-Sp0	42	1
13	293	(6S)Galb1-4(6S)GlcNAcb-Sp0	41	5
14	295	Neu5Aca2-3Galb1-4(Fuca1-3)GlcNAcb1-6(Galb1-3)GalNAca-Sp14	41	2
15	24	(3S)Galb1-4(Fuca1-3)(6S)Glc-Sp0	41	2
16	135	Neu5Aca2-6(Galb1-3)GalNAca-Sp8	41	1
17	44	(6S)Galb1-4GlcNAcb-Sp8	40	5
18	34	(3S)Galb1-4(6S)GlcNAcb-Sp0	39	2
19	22	6S(3S)Galb1-4(6S)GlcNAcb-Sp0	39	3
20	40	(4S)Galb1-4GlcNAcb-Sp8	39	6
91	246	Neu5Aca2-3Galb1-3GlcNAcb-Sp0	24	3
98	436	(6S)Galb1-3GlcNAcb-Sp0	24	5
107	250	Neu5Aca2-3Galb1-4(Fuca1-3)GlcNAcb-Sp0	23	5
120	251	Neu5Aca2-3Galb1-4(Fuca1-3)GlcNAcb-Sp8	21	3
270	149	Galb1-3GlcNAcb-Sp0	10	4
318	41	(6P)Mana-Sp8	7	3
325	236	Neu5Aca2-3Galb1-3(Fuca1-4)GlcNAcb-Sp8	6	3
329	128	Galb1-3(Fuca1-4)GlcNAc-Sp0	6	3
340	102	Gala1-3(Fuca1-2)Galb1-3GlcNAcb-Sp8	6	3
417	129	Galb1-3(Fuca1-4)GlcNAc-Sp8	3	1

IgG Affinities (50 μg/mL)

Rank	ID	Glycan	RFA	SD
1	28	(3S)Galb1-3(Fuca1-4)GlcNAcb-Sp8	58890	3160
2	484	(3S)Galb1-3(Fuca1-4)GlcNAcb-Sp0	44494	1393
3	30	(3S)Galb1-3GlcNAcb-Sp0	24516	16706
4	26	(3S)Galb1-4(6S)Glcb-Sp0	12734	1905
5	31	(3S)Galb1-3GlcNAcb-Sp8	12514	2166
6	27	(3S)Galb1-4(6S)Glcb-Sp8	6189	1011
7	34	(3S)Galb1-4(6S)GlcNAcb-Sp0	3287	217
8	29	(3S)Galb1-3GalNAca-Sp8	3269	246
9	35	(3S)Galb1-4(6S)GlcNAcb-Sp8	2626	303
10	545	GlcNAcb1-3Galb1-4GlcNAcb1-6(GlcNAcb1-3Galb1-4GlcNAcb1-2)Mana1-6(GlcNAcb1-3Galb1-4GlcNAcb1-2Man a1-3)Manb1-4GlcNAcb1-4GlcNAc-Sp24	1442	651
11	38	(3S)Galb-Sp8	1438	118
12	531	GlcNAcb1-3Galb1-4GlcNAcb1-2Mana1-6(GlcNAcb1-3Galb1-4GlcNAcb1-2Mana1-3)Manb1-4GlcNAcb1-4GlcNAcb-Sp12	1079	116
13	45	(6S)Galb1-4(6S)Glcb-Sp8	978	48
14	537	Galb1-4GlcNAcb1-3Galb1-4GlcNAcb1-3Galb1-4GlcNAcb1-2Mana1-6(Galb1-4GlcNAcb1-3Galb1-4GlcNAcb1-3Galb1-4GlcNAcb1-2Mana1-3)Manb1-4GlcNAcb1-4GlcNAcb-Sp12	796	250
15	293	(6S)Galb1-4(6S)GlcNAcb-Sp0	654	37
16	22	6S(3S)Galb1-4(6S)GlcNAcb-Sp0	629	20
17	84	(3S)Galb1-4(Fuca1-3)Glcb-Sp0	433	28
18	37	(3S)Galb1-4GlcNAcb-Sp8	430	24
19	25	(3S)Galb1-4Glcb-Sp8	424	32
20	562	Galb1-4GlcNAcb1-3Galb1-4GlcNAcb1-6(Galb1-4GlcNAcb1-3Galb1-4GlcNAb1-2)Mana1-6(Galb1-4GlcNAcb1-3Galb1-4GlcNAcb1-2Mana1-3)Manb1-4GlcNAcb1-4(Fuca1-6)GlcNAcb-Sp24	277	84
57	44	(6S)Galb1-4GlcNAcb-Sp8	36	5
82	436	(6S)Galb1-3GlcNAcb-Sp0	25	3
96	246	Neu5Aca2-3Galb1-3GlcNAcb-Sp0	22	2
160	41	(6P)Mana-Sp8	17	5
247	128	Galb1-3(Fuca1-4)GlcNAc-Sp0	13	2
251	250	Neu5Aca2-3Galb1-4(Fuca1-3)GlcNAcb-Sp0	13	1
305	251	Neu5Aca2-3Galb1-4(Fuca1-3)GlcNAcb-Sp8	12	1
311	236	Neu5Aca2-3Galb1-3(Fuca1-4)GlcNAcb-Sp8	11	2
324	149	Galb1-3GlcNAcb-Sp0	10	1
487	129	Galb1-3(Fuca1-4)GlcNAc-Sp8	5	1
488	150	Galb1-3GlcNAcb-Sp8	5	1

IgM Affinities (5 μg/mL)

Rank	ID	Glycan	RFA	SD
1	30	(3S)Galb1-3GlcNAcb-Sp0	53365	6505
2	293	(6S)Galb1-4(6S)GlcNAcb-Sp0	52774	4529
3	28	(3S)Galb1-3(Fuca1-4)GlcNAcb-Sp8	52578	6381
4	26	(3S)Galb1-4(6S)Glcb-Sp0	44761	2990
5	34	(3S)Galb1-4(6S)GlcNAcb-Sp0	37881	1585
6	484	(3S)Galb1-3(Fuca1-4)GlcNAcb-Sp0	36012	2788
7	31	(3S)Galb1-3GlcNAcb-Sp8	35173	1694
8	27	(3S)Galb1-4(6S)Glcb-Sp8	34384	4456
9	45	(6S)Galb1-4(6S)Glcb-Sp8	34222	2681
10	22	6S(3S)Galb1-4(6S)GlcNAcb-Sp0	34066	1021
11	35	(3S)Galb1-4(6S)GlcNAcb-Sp8	30024	2286
12	38	(3S)Galb-Sp8	28471	2188
13	29	(3S)Galb1-3GalNAca-Sp8	28315	1943
14	25	(3S)Galb1-4Glcb-Sp8	6633	967
15	217	(3S)Galb1-4(Fuca1-3)(6S)GlcNAcb-Sp8	6250	849
16	37	(3S)Galb1-4GlcNAcb-Sp8	2454	405
17	84	(3S)Galb1-4(Fuca1-3)Glcb-Sp0	2192	142
18	437	(6S)Galb1-3(6S)GlcNAc-Sp0	390	15
19	36	(3S)Galb1-4GlcNAcb-Sp0	372	52
20	187	GlcNAcb1-6(GlcNAcb1-4)GalNAca-Sp8	239	14
27	44	(6S)Galb1-4GlcNAcb-Sp8	44	2
60	436	(6S)Galb1-3GlcNAcb-Sp0	23	4
85	246	Neu5Aca2-3Galb1-3GlcNAcb-Sp0	19	4
124	250	Neu5Aca2-3Galb1-4(Fuca1-3)GlcNAcb-Sp0	15	2
237	41	(6P)Mana-Sp8	8	1
314	128	Galb1-3(Fuca1-4)GlcNAc-Sp0	6	1
317	236	Neu5Aca2-3Galb1-3(Fuca1-4)GlcNAcb-Sp8	6	1
318	251	Neu5Aca2-3Galb1-4(Fuca1-3)GlcNAcb-Sp8	6	2
329	149	Galb1-3GlcNAcb-Sp0	5	2
423	129	Galb1-3(Fuca1-4)GlcNAc-Sp8	4	2
464	102	Gala1-3(Fuca1-2)Galb1-3GlcNAcb-Sp8	3	1
