# Supplementary figures and images for: mAb Das-1 recognizes 3’-Sulfated Lewis A/C, which is aberrantly expressed during metaplastic and oncogenic transformation of several gastrointestinal Epithelia
Source: PLoS One. 2021 Dec 15;16(12):e0261082. doi: 10.1371/journal.pone.0261082 (PMC8673611; doi:10.1371/journal.pone.0261082)

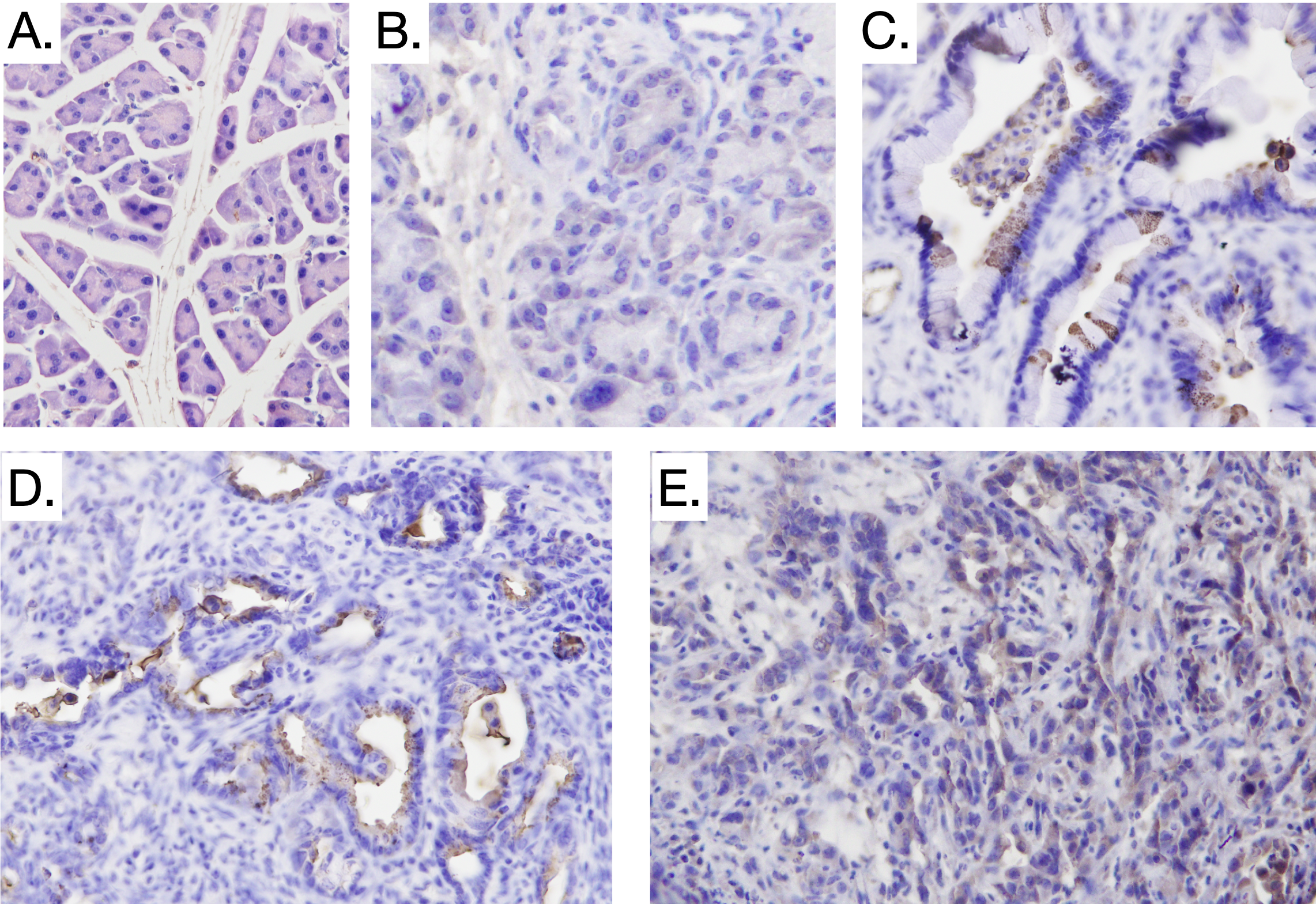

Supplement: S2 Fig — Das-1 is not reactive to (A) normal pancreata or (B) acinar-to-ductal metaplasia; however, demonstrates a (C) variegated reactivity towards high-grade Pan-IN, which becomes confluent in (D) pancreatic ductal carcinoma (PDAC) and (E) invasive PDAC. This pattern phenocopies what we have observed our survey of human pancreatic cancer progression (Das et al. (2021) Human Pathology 111: 36–44). (TIFF) [file pone.0261082.s002.tiff]

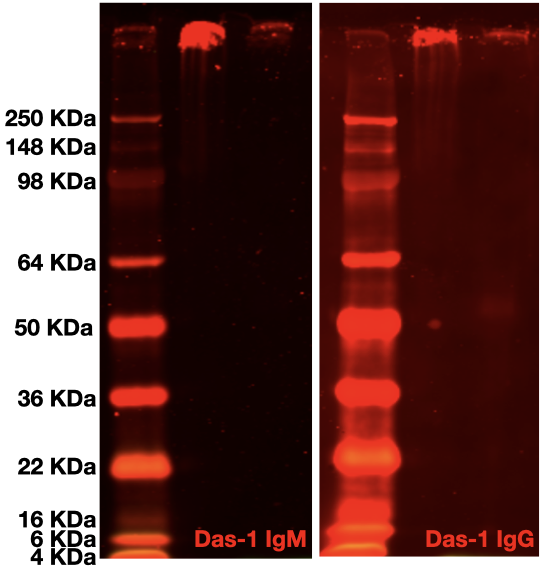

Supplement: S1 Raw images — (TIFF) [file pone.0261082.s006.tiff]
